# Microneedle Sensors for Ion Monitoring in Plants. One Step Closer to Smart Agriculture

**DOI:** 10.1021/acssensors.5c01215

**Published:** 2025-07-03

**Authors:** Qianyu Wang, Águeda Molinero-Fernandez, José Ramón Acosta Motos, Gastón A. Crespo, María Cuartero

**Affiliations:** † Department of Chemistry, KTH Royal Institute of Technology, Teknikringen 30, Stockholm SE-114 28, Sweden; ‡ UCAM-SENS, Universidad Católica San Antonio de Murcia, UCAM HiTech, Avda. Andre’s Herna’ndez Ros 1, Murcia 30107, Spain; § Plant Biotechnology for Food and Agriculture Group (BioVegA), Universidad Católica San Antonio de Murcia (UCAM), Murcia 30107, Spain; ∥ Plant Biotechnology, Agriculture and Climate Resilience Group, Associate Unit of R&D+i CSIC-UCAM, Murcia 30100, Spain

**Keywords:** wearable sensor, sap analysis, ion signaling, electrochemical sensor, plant stress

## Abstract

As global demand for food rises and agricultural systems face unprecedented stress from environmental challenges, understanding the role of ions (i.e., key nutrient components) in crop productivity has never been more critical. Unfortunately, current tools for ion analysis in plants rely on destructive sap collection that fails to capture the dynamic changes in ionic concentrations. On the other hand, noninvasive optical methods lack practicality for field applications due to their reliance on expensive equipment and complex operational procedures. Recent advancements in microneedle (MN) sensing technology have demonstrated significant potential for real-time monitoring of plants’ health by enabling the direct detection of various important biomarkers, including but not limited to ions. By offering a minimally invasive approach, MN sensors allow continuous in-planta monitoring with precise penetration into plant tissues, ensuring natural growth remains undisturbed. However, the application of MN sensors, especially for in vivo ion measurement, is still in its very early stage. Herein, we delve into the technological potential and application avenues of plant MN sensors, with a focus on tailoring sensor designs to meet the specific requirements of various plant growth environments and analytical performances for ion detection. This perspective paper also introduces the essential relevance of ion levels in plants, provides a comprehensive assessment of existing ion detection methods, and identifies key challenges associated with achieving effective in planta monitoring. Notably, we highlight the potential of MN sensors as a transformative approach for unveiling plant stress responses, optimizing crop yields, and fulfilling diverse roles that bridge the fields of precision agriculture and plant science research.

## Graphical abstract

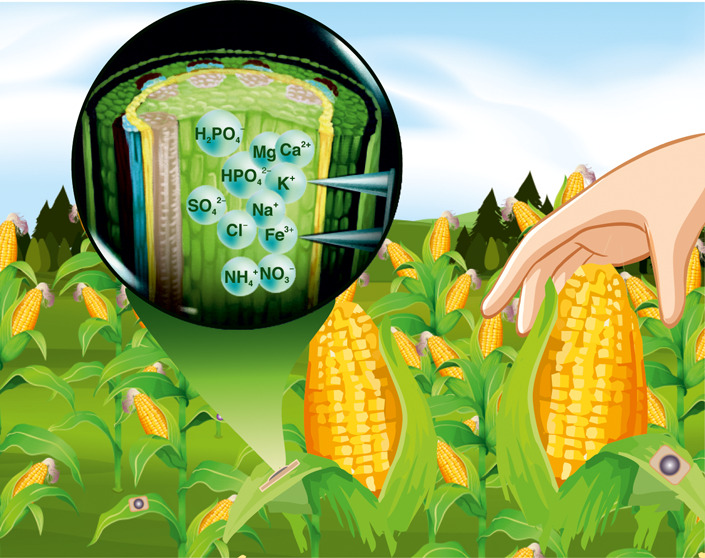

The global population is projected to reach approximately 9.8 billion by the mid-21st century.^1^ To meet the increasing demand for food, production must increase by 70% to 100% compared to current levels.^2^ Climate change further complicates this scenario, as shifting weather patterns, extreme events, and temperature changes can affect crop yields and agricultural productivity. To achieve the sustainable food goal, it is essential to bridge the gap between the current agricultural output and the theoretical “yield potential” (i.e., the maximum yield attainable under optimal conditions).^3^

Among the variety of emerging technologies, such as genetically encoded crops and controlled environment agriculture, proposed for transforming agriculture, precision agriculture stands out due to its transformative potential. By leveraging advanced tools like a new era of sensors to monitor crop health status, farmers will be enabled to make faster corrective decisions, reduce waste, and maximize yield. In particular, chemical sensors related to early stress biomarkers, such as ions, are especially attractive.^1^ To date, the main developments in this area have been focused on soil monitoring;^4^ however, this alone does not provide a complete or accurate assessment of a plant’s health status, which highlights the need for new sensors that can directly assess plant stress and, overall, well-being. Since ions are primarily located in the plant sap, their analysis requires methods capable of reaching this internal fluid. To analyze sap, conventional approaches commonly involve physical or chemical processes to release the sap for its subsequent ionic content analysis in centralized laboratory-based instruments or ion-selective electrodes (ISEs).^5,6^ Due to the extraction step, these methods cause significant damage to the plants, ranging from localized injury to total loss. Additionally, this method is inherently limited to capturing discrete data, hindering the monitoring of transient and long-distance ionic signals, such as calcium (Ca^2+^) signaling,^7,8^ and failing to capture these dynamic and time-sensitive events.^9^

Alternatively, optical methods have been proposed, such as red-green-blue (RGB) imaging,^10^ hyperspectral imaging,^11^ and the use of nondestructive nanosensors,^12^ enable noninvasive and real-time assessments of plant health. Nevertheless, these imaging techniques provide only indirect insights into ionic composition via interpreting the optical signatures exhibited by plants. Thus, these methods are highly susceptible to background noise from ambient light interferences, which substantially compromises their sensitivity, selectivity, and overall accuracy.^13^ Also, sophisticated instruments and procedures such as fluorescent protein incorporation in the plant’s system by injection or genetic encoding are required, which are unfeasible for real field applications.^14^ The safety of the (nano)­materials employed for its development is still unclear, and specific regulatory safety standards are needed before its implementation.^15^ So far, these optical sensors have only been tested in highly controlled laboratories, leaving their practical utility in agricultural contexts largely unvalidated.

Electrochemical methods have also been considered for biomarkers monitoring in plants, owing to their high sensitivity, selectivity, rapid response, and portability.^16–18^ Recently, plant-wearable sensors have been developed to monitor various physiological and environmental parameters, such as plant growth (e.g., leaf and stem elongation^19,20^ ), microclimate conditions,^21,22^ and key molecules, including hormones,^23^ volatile organic compounds,^13^ metabolites,^24,25^ and pesticides.^26^ These sensors are designed to be placed on the surface of the plant and, as such, they are developed in a planar and flexible format. Notably, when planar flexible sensors are implemented for sap analysis, they face significant challenges due to two primary limitations: (1) the insufficient volume of sap released through stomata, which hinders consistent analysis, and (2) the absence of effective strategies to actively induce sufficient sap excretion for reliable measurement.

To overcome the challenges associated with sap collection and analysis, microneedle (MN) sensing platforms offer a disruptive approach to in-planta analysis.^27^ Effectively, MN-based sensors are widely recognized for their minimally invasive nature, enabling effortless penetration of the plant epidermis. This process provides direct access to and interaction with sap. Truly, the penetration step has been shown not to hinder normal plant growth, as evidenced by several studies.^28–30^ Recently, the application of MN sensors for plant health monitoring has gained significant attention, demonstrating the potential and justifying the rapid development of this emerging field.^29–33^

This perspective paper focuses on recent advancements in MN sensors specifically designed for ion monitoring. Figure 1 illustrates how it is partitioned into four sections. As an initial context, the significance of ion monitoring in plants is discussed. Then, the methodology for monitoring ions in plants that is currently in use is described. Subsequently, a concise literature review of recent examples of MNs sensors in plants is presented, as well as their potential for ion monitoring. The final section addresses the critical knowledge shortcomings, challenges, and outlook on future development and impact research on this subject.

**Figure 1:**
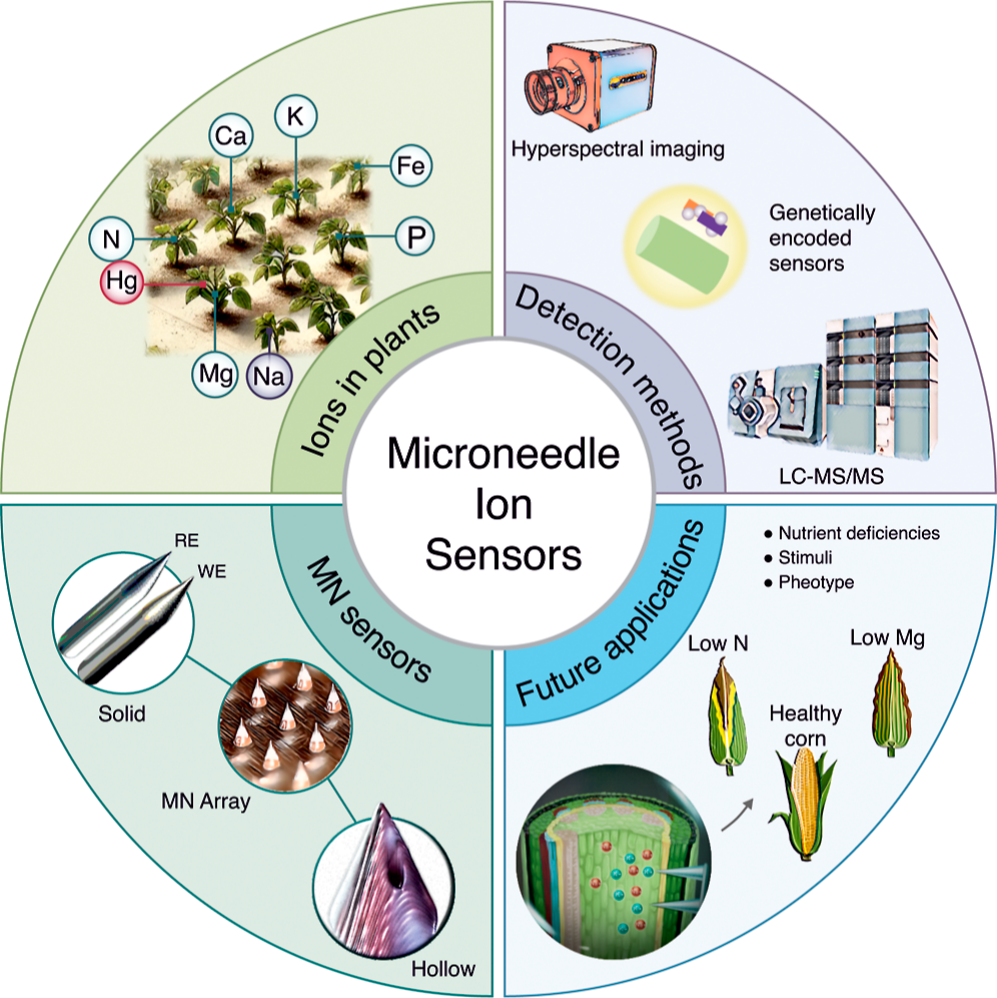
A summary figure depicting the four primary discussion areas of this perspective paper: the importance of ions in plants, current detection methodologies, various microneedle sensor designs, and prospective future applications.

## Ions as Early Stress Biomarkers

Ions are involved in numerous physiological processes in plants (Figure 2), from providing essential nutrients to chemical signaling. Each ion uniquely contributes to plant life, either independently regulating specific physiological processes or working synergistically to drive complex biochemical interactions in all the parts of the plants (from roots to fruits). Just to number a few, ion functions include signal transduction to environmental stimuli,^34^ facilitating enzyme activation (e.g., K^+^ is required for the activation of over 60 enzymes),^35^ maintenance of the osmotic balance,^36^ stabilizing membrane potential.^37^ More in detail, macronutrients, such as nitrate ions (NO_3_^–^), and phosphate ions (PO_4_^3–^) are essential for protein synthesis and plant growth.^38,39^ Potassium ions (K^+^), which rank among the most essential ions in plants, fulfill a wide range of critical functions and are maintained at cytoplasmic concentrations ranging from 100 to 200 mM.^40^ Ionic nutrients such as magnesium (Mg^2+^) and iron (Fe^3+^) have specialized roles in photosynthesis, serving as integral components of the chlorophyll molecule. To show more clearly its significance in plant functions and plant organ singularities, a summary of the key ions classified by their proportion in plants (i.e., macro- and micronutrients) is provided in Tables 1 and 2. Note that ion concentrations vary significantly between plant species and growth stages, making standardization difficult. While most typical ranges are included in the tables, specific cases should be studied individually.

**Figure 2:**
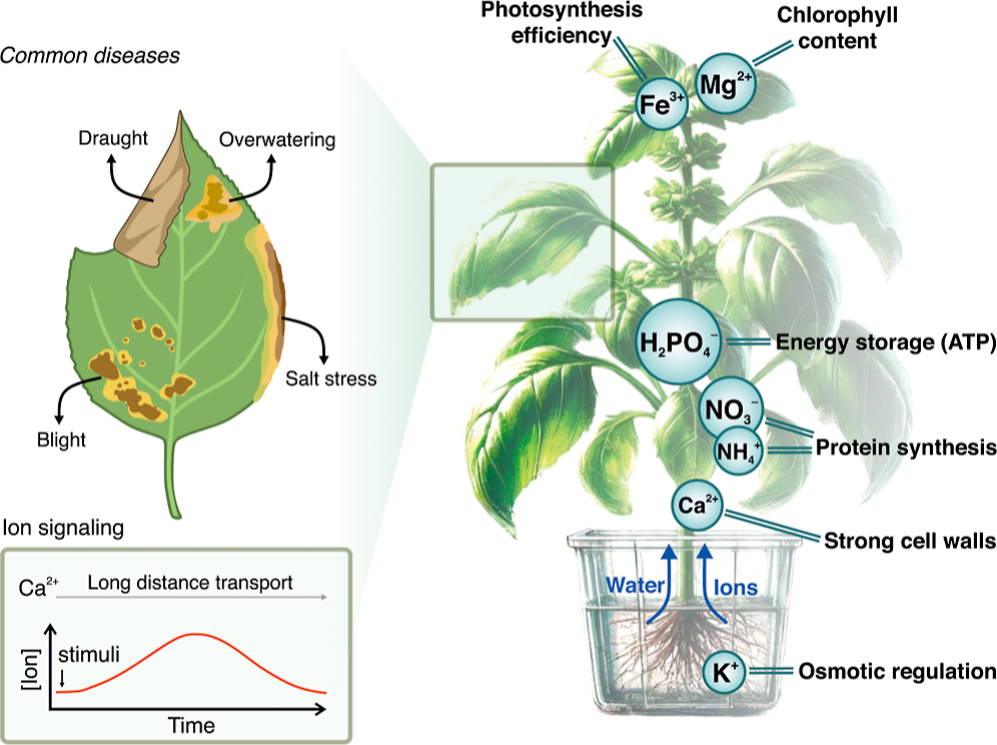
Essential roles of ions in regulating physiological processes in plants.

**Table 1:** Main Ionic Macronutrients in Plant Homeostasis

ion	importance	plant organ	role in the plant	typical ranges	ref
K^+^	high	stem	osmotic regulation and water and nutrient transportation	50–150 mM	^48^
		leaves	enzyme activation, photosynthesis	1 mM	^49^
		root	maintains cell turgor and regulates root pressure during nutrient uptake	10–30 mM	^50^
N (NH_4_^+^, NO_3_^–^)	high	leaves	major component of chlorophyll, essential for photosynthesis	20–50 mM	^49^
		root	enhances root growth and influences root architecture	5–10 mM	^51^
P (H_2_PO_4_^–^, HPO_4_^2–^)	high	stem	energy transfer (ATP), signaling pathways	5–15 mM	^52^
		root	affects root elongation and nutrient absorption efficiency	1–3 mM	^53^
Ca^2+^	high	leaves	structural component of cell walls, signaling	3–10 mM	^54^
		root	supports root tip growth and ion transport	2–5 mM	^55^
Mg^2+^	medium	leaves	central atom in chlorophyll; enzyme cofactor in photosynthesis	1–3 mM	^56^
		root	enhances root nutrient uptake and stress tolerance	0.5–1 mM	^57^
S (SO_4_^2–^)	high	leaves	component of amino acids (cysteine, methionine), proteins, and coenzymes	1–2 mM	^58^
		root	enhances root metabolism and enzyme activation	0.5–1 mM	^59^
Na^+^	medium	leaves	maintains osmotic potential in halophytes, substitutes for K^+^ under stress	1–5 mM	^60^
		root	facilitates nutrient uptake in salt-tolerant plants	0.5–1 mM	^61^
Cl^–^	medium	leaves	essential for photosynthesis (water-splitting reaction)	0.1–0.5 mM	^62^
		roots	aids in maintaining charge balance and osmotic pressure	0.05–0.2 mM	^63^

**Table 2:** Main Ionic Micronutrients in Plant Homeostasis

ion	importance	plant organ	role in the plant	typical ranges	ref
Fe^2+^, Fe^3+^	high	leaves	essential for chlorophyll synthesis and electron transport	20–100 μM	^64^
		root	critical for root respiration and iron uptake mechanisms	10–30 μM	^65^
Zn^2+^	medium	leaves	activates enzymes, regulates photosynthesis	10–50 μM	^66^
		root	promotes root elongation and hormonal balance	5–20 μM	^67^
Mn^2+^	medium	leaves	involved in water splitting during photosynthesis	20–200 μM	^68^
		root	essential for root structure and nutrient transport	10–30 μM	^69^
Cu^2+^	low	leaves	cofactor in electron transport and oxidative stress enzymes	5–20 μM	^70^
		root	important for root lignification and respiration	2–10 μM	^71^
B (H_3_BO_3_)	medium	leaves	essential for cell wall stability and sugar transport	20–100 μM	^72^
		root	aids in root elongation and cell division	5–10 μM	^73^
Mo (MoO_4_^2–^)	low	leaves	cofactor in nitrogen assimilation (nitrate reductase)	0.1–1 μM	^74^
		root	facilitates nitrogen uptake in legumes	0.05–0.2 μM	^72^

To achieve optimal agricultural productivity, plants require a consistent supply of at least 14 macro- and micronutrients, which are sourced from the soil or fertilizers.^41^ Repeated fertilization is often necessary to address deficiencies from insufficient nutrient absorption by plants.^42^ On the other hand, excessive fertilization will pose significant risks, such as nutrient imbalances, salt accumulation, and root damage, all of which impede plant growth and development.^43^ Prolonged exposure to some ions such as Na^+^ can be lethal to most plants.^44^ In this context, although some sensors can provide real-time data on ion concentrations in soil, they do not accurately reflect the situation along the plant due to the complexity, plant species variability, and the dynamic nature of nutrient availability.^4,9^

Truly, ion dynamics can enhance agricultural production and elucidate plant responses to environmental stressors. Changes in ion concentrations and their ratios imply biotic stress (resulting from diseases, insects, etc.) or abiotic stress (stemming from environmental conditions such as dryness, salinity, etc.).^45^ For example, major stressors, such as infections and physical injuries, induce Ca^2+^ signaling in most plants.^7^ Indeed, ions also hold significant potential as early biomarkers of plant diseases. Furthermore, nutrient levels in plants directly influence their tolerance and resistance to pathogens.^46^ The K^+^ supplementation has been proved to reduce the overall incidence of disease by 66%, providing protection against a wide range of pathogens, including fungi, bacteria, and viruses.^47^ However, plant ionomics (i.e., the study of mineral nutrients and trace elements in plants) remains relatively underdeveloped, particularly in ion responses to plant pathogens. And its progress is mainly limited until now by the lack of high-throughput tools for real-time monitoring of ion dynamics, as current methodologies predominantly depend on laboratory-based equipment.

## Current Analytical Methods for ION Detection

To develop new reliable and suitable MN sensors (or any other tool indeed) for precision agriculture, it is crucial to first understand the structure of plants. Starting from the more external to the most internal part, the plant’s epidermis serves as a tough, often rigid, outermost layer composed primarily of tightly packed cells, forming a robust protective barrier. This layer shields internal tissues from physical damage and dehydration. In vascular plants, the xylem and phloem constitute essential tissues responsible for the transport of water, nutrients, and photosynthates. The xylem primarily facilitates the movement of water and minerals from the roots to the leaves, while the phloem distributes sugars and other organic compounds throughout the plant (Figure 3a).^17^ In the leaf, the xylem and phloem run parallel to each other, forming together the vascular bundle. Ions are commonly present in the fluids transported by these vascular tissues, commonly known as plant sap. This constitutes a significant challenge for conventional methods, which struggle to penetrate the plant’s epidermal barrier without causing damage.

**Figure 3:**
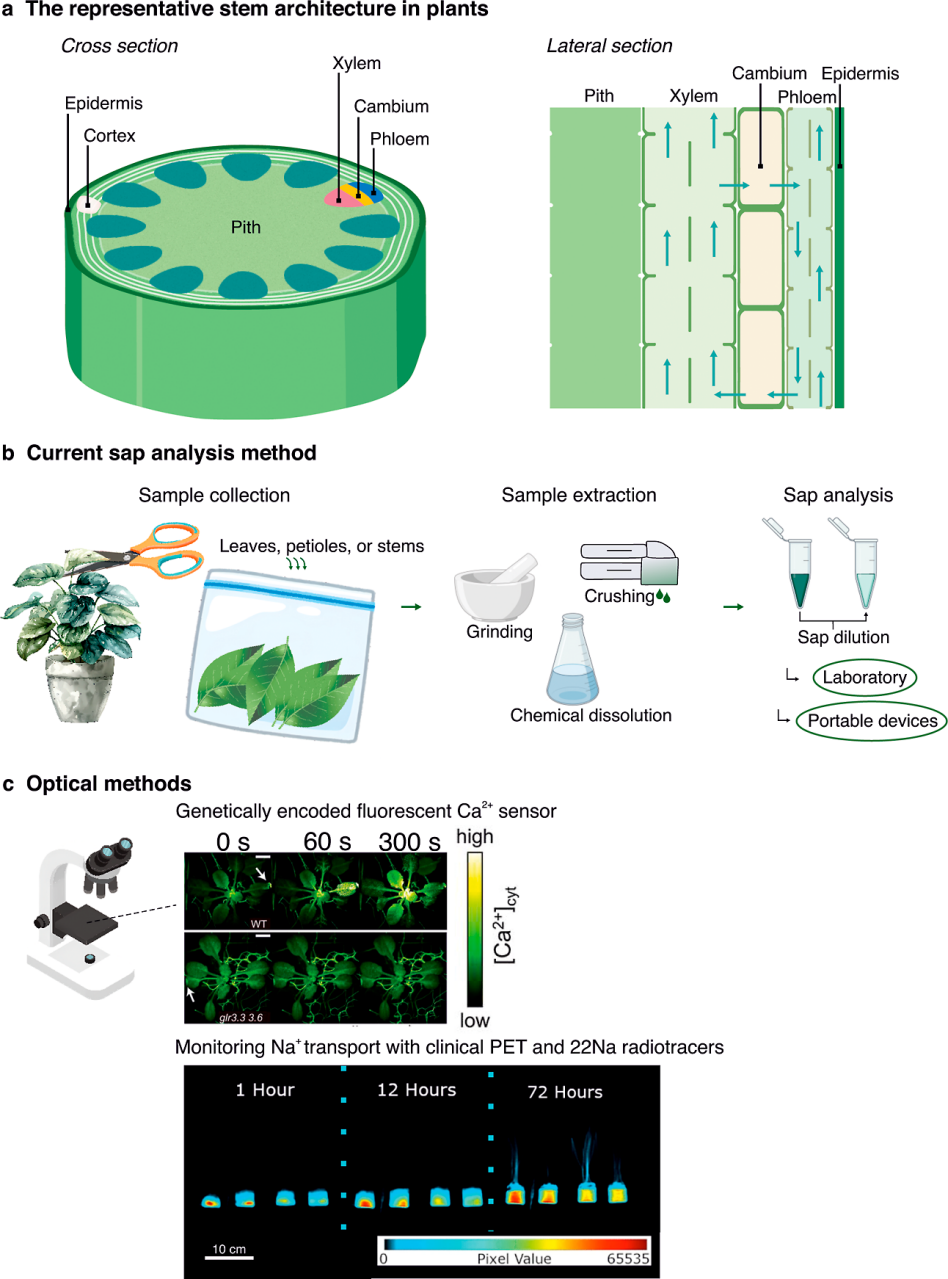
(a) Cross-sectional and lateral section views of a dicot plant stem. (b) Overview of sap analysis workflow: collection, extraction, and analysis. (c) Optical techniques for in vivo ion monitoring in plants. Adapted with permission from refs 86 and 87 Reproduced from ref 86. Copyright 2018 American Association for the Advancement of Science. Adapted from ref 87. Available under CC-By 4.0. Copyright 2021 Ruwanpathirana et al.

## Conventional Methods

The traditional analytical workflow requires the collection of plant tissue samples, which are obtained from stems or leaves. These samples are mechanically homogenized and subjected to acid digestion to solubilize ions for subsequent analysis (Figure 3b). For this purpose, commonly employed instruments include atomic absorption spectrometers (AAS),^75^ inductively coupled plasma optical emission spectrometers (ICP-OES),^76^ liquid chromatography-tandem mass spectrometry (LC–MS/MS),^77^ and ion chromatography (IC).^78^ Equipment-based sap analysis is widely considered the gold standard in plant physiological research due to its capability to deliver highly accurate and reliable data. However, the conventional methods need specialized training for proper operation and rely heavily on field sampling and laboratory-based analysis, which often introduces delays and disrupts the continuity of data collection. Their inherently destructive nature compromises plant health and tissue integrity. These limit the practicability of on-site testing and impede continuous, long-term measurements. Consequently, opportunities for early disease detection may be missed, and potential inaccuracies may be further exacerbated by risks such as sample evaporation and contamination during transportation.

## Optical Methods

Optical sensors can circumvent numerous issues commonly linked to whole-plant sensors because of their noncontact method for light measurements. Consequently, numerous optical techniques have been suggested for the noninvasive identification of chemical components within deeper plant tissues. For example, unmanned aerial vehicles (UAVs) outfitted with red–green–blue (RGB) imaging technology have been employed in field trials to remotely identify nitrogen deficit in maize leaves.^79^ Hyperspectral imaging (HSI) is utilized to mitigate the problem of low resolution associated with the RGB imaging technique.^80^ Inconveniently, the accuracy of detection is mostly affected by environmental factors, including solar illumination and meteorological conditions. Then, optimal, uniformly dispersed illumination can solely be attained in controlled settings, hence constraining the practical applicability of these imaging techniques.^81^ A further drawback is that ion levels are inferred implicitly through visual indicators, such as alterations in leaf morphology, rather than being explicitly quantified by measuring their concentrations.^10^ Thus, imaging approaches suffer significant interference from leaf surface angles, distances, and interleaf reflectance. These conditions may induce distortions in spectral signatures, undermining the precision and dependability of the data.^82^

X-ray fluorescence spectroscopy (XRF) is a noninvasive and portable technique that employs high-energy particles or X-ray beams with short wavelengths and high frequencies to detect elemental compositions, such as zinc and manganese, in the deeper layers of plant tissues.^83^ Yet, the use of X-rays inherently results in radiation-induced damage to living biological samples. Developing a standardized protocol that can be universally applied to various sample types remains challenging due to the complex interplay between X-ray flux, dosage, and sample damage.

Genetically encoded optical (nano)­sensors offer a promising method for the real-time observation of dynamic processes in plants (Figure 3c). These sensors employ specially designed proteins that engage with target analytes, resulting in observable alterations in fluorescence signals. Optical Ca^2+^ sensors typically utilize calmodulin, a Ca^2+^-binding protein that experiences changes in structure upon contact with Ca^2+^ ions. This type of sensors is generally inserted into plants by diverse delivery methods.^84^ Then, fluorescence microscopy is used to visualize and map ion concentrations within the plants under controlled laboratory conditions.^85^ Genetically encoded biosensors typically necessitate apparatus like confocal or fluorescent microscopes. This limits their portability for application beyond laboratory environments. Their analytical performances are influenced by environmental parameters, including temperature, humidity, and light conditions.

## Microneedle Sensors for In-Planta Ion Monitoring

A method for penetrating the plant’s epidermal barrier and accessing interior fluids involves the utilization of microneedle sensors. MNs resemble intradermal needles but are considerably smaller, generally averaging approximately 1000 μm in length. Originally proposed as a substitute for transdermal drug administration, MNs have recently attracted considerable interest as sensor elements (or carriers) in multiple fields. Their primary use is in healthcare applications through skin interstitial fluid analysis, whereas their implementation for in-plant monitoring remains limited.^88^ Accordingly, this signifies a revolutionary frontier in plant science, marked by extensive research potential and many applications.

To the best of our knowledge, all investigations on MN sensors for the ongoing assessment of plant health have utilized electrochemical approaches, owing to their rapid response, high sensitivity, and exceptional selectivity. Moreover, its intrinsic capacity for size reduction facilitates simple incorporation onto printed circuit boards (PCBs) and enables wireless data transfer through compact, portable devices for in vivo monitoring. This permits on-site evaluations of the plant status and hence, the adoption of early corrective actions prior to any irreversible plant damage.^89^

## Requirements for the Microneedle Sensor Design

Beyond obvious analytical requirements, two critical factors must be considered for effective MN sensor design: (i) the sensor’s resistance to penetration and (ii) its ability to access plant fluids without inflicting tissue harm. Parameters like length, diameter, material, form, tip angle, and insertion force are critical for enhancing resistance to plant insertion in the development of a reliable device. Those aspects have been widely explored for healthcare, and in some cases extrapolated to other fields. However, plants present inherent singularities that cannot be overpassed. MN sensors are constructed with tip diameters generally varying from 5 to 80 μm, even after the incorporation of sensing layers.^88^ Importantly, the lengths should be accurately customized to suit diverse plant tissues (e.g., pith, xylem, phloem), organs (e.g., roots, stems, leaves, fruits), and numerous plant species. MN geometries may assume either pyramidal or cylindrical forms may enable seamless penetration into plant tissues. Indeed, recent literature has showed that a basic finger press exerting around 20–40 N of force is adequate for the insertion of MNs of different configurations.^29,30^

To examine the potential damage resulting from MN implantation, postpuncture assessments of wound dimensions and plant health indicators should be conducted. The wound dimensions generally correspond with the specified design parameters of the MNs, often limited to a few hundred microns.^29^ A minimal disruption is anticipated to exert negligible consequences on the plant’s structural integrity. As such, both the leaves and stems of the plants observed after 15 to 30 days post-MN insertion demonstrated normal growth and development.^28,30^ Preliminary tests revealed a wound-healing effect on tomato leaves for 3 days following puncture with microneedle arrays (Figure 4a). Aloe vera leaves exhibited a full recovery process over 15 days (Figure 4a). Simultaneously, basil stems exhibited rapid healing, with scabbing observable at 10 min after MN insertion (Figure 4b).^28^ In all these instances, the plants may grow normally following microneedle insertions. These discoveries demonstrate how plants inherently heal damage incurred during growth, such as impacts from debris, hailstorms, avian pecks, or incisions from agricultural implements and trimming. Indeed, plants have evolved highly efficient mechanisms for self-repair following damage.^90^ Notably, researchers have utilized statistical data to look at the impact of MNs on plants. Parameters such as stem diameter, nitrogen content, and chlorophyll content are measured several days postinsertion.^32^ The results once more confirmed that MNs exert negligible influence on the regular growth of plants.

**Figure 4:**
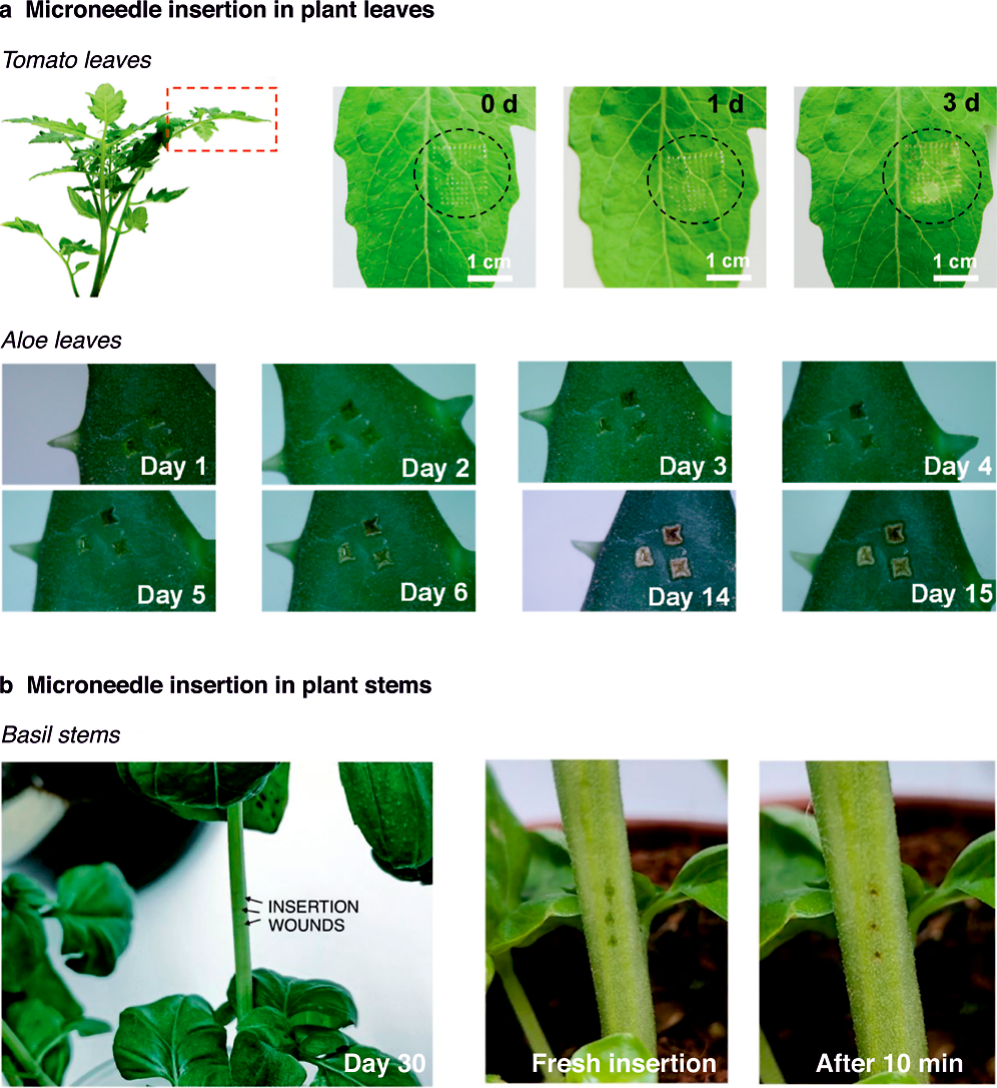
Wound healing following microneedle insertions in (a) plant leaves,^30,32^ reproduced from ref 30 Copyright 2024 Elsevier, and reproduced from ref 32. Copyright 2024 Wiley, and (b) plant stems.^28^ Reproduced from ref 28. Available under CC-By 4.0. Copyright 2024 Wang et al.

## First Examples of Microneedle Sensors for In-Planta Monitoring

Various materials, such as stainless steel and polymers, can be utilized to fabricate solid, coated, or hollow MNs for in-plant sensing. Each MN type has distinct properties, advantages, and limitations, which are examined in the subsequent section.

## Solid Microneedles

Figure 5a illustrates a solid MN produced by the mold casting technique, a common method to produce solid polymeric MN arrays for healthcare applications.^91^ The polymer formulation is placed into a mold commonly made from polydimethylsiloxane (PDMS).^91^ The properties of the produced MNs are significantly influenced by the selection of materials. Researchers utilized a hydrogel made of poly­(methyl vinyl ether-alt-maleic acid) cross-linked with polyethylene glycol to develop MN arrays, with overall dimensions of the individual needles of 700 μm in height and 260 μm in bottom diameter. When MNs were inserted into the plant, they quickly swelled, absorbing 2.20 ± 0.30 mg of sap in only 1 min (Figure 5a).^32^ Then, the extracted sap is analyzed by a portable photoelectric colorimeter. This strategy is an interesting alternative to the destructive sap extraction methods. However, continuous signals cannot be obtained and for long-term data collection, serial insertions using different MN patches would be necessary.

**Figure 5:**
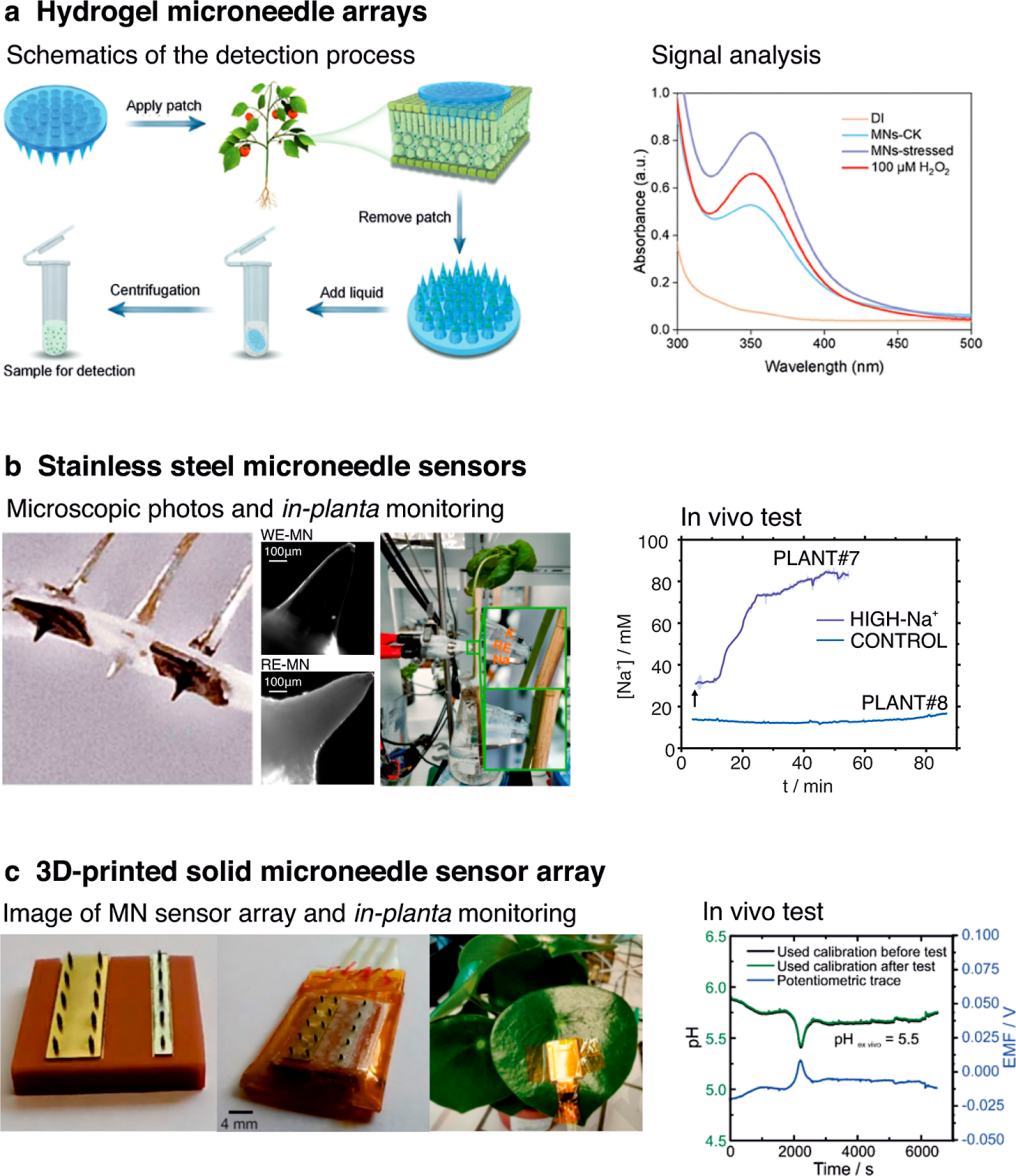
Solid MN sensors for in-planta monitoring. Different manufacturing techniques are used: (a) mold casting hydrogel MN array for sap extraction,^32^ reproduced from ref 32. Copyright 2024 Wiley, (b) coated stainless steel MN sensors for K^+^ and Na^+^ detection,^28^ reproduced from ref 28. Available under CC-By 4.0. Copyright 2024 Wang et al. and (c) 3D-printed MN arrays for in vivo pH monitoring in plants. Reproduced from ref 33. Copyright 2024 Elsevier.

A coating approach to incorporate sensing capabilities to solid MNs has been recently implemented and demonstrated for in-planta MNs sensors. Commercially available solid stainless steel MNs of medical grade were transformed into ion-selective sensors able of monitoring K^+^ and Na^+^ ions in plants (Figure 5b).^28^ Both ion-selective MNs (i.e., K^+^, and Na^+^) are combined with a reference MN to obtain continuous signals with minimal tissue damage due to their dimensions (625 μm height and 300 μm in bottom). Using this approach, salt stress was directly monitored, providing high spatiotemporal resolution of Na^+^ concentration. Notably, a critical challenge for externally modified MN sensors (such as those obtained with the coating protocol) is to ensure consistent analytical performance after insertion, since the sensing element are in direct contact with the tissues and kind of “unprotected” during the insertion and extraction processes. A widely adopted process to address this issue is the application of an external polymer membrane, such as one made of polyurethane. This polymer layer serves as a protective barrier while providing robust mechanical support to the sensor and smoothing the insertion.^28,91^

Another way of fabricating solid MNs is by using three-dimensional (3D) printing technology. Then, the fabricated MNs can be equipped with sensing capabilities by coating the sensing layer, as just discussed. This technique provides great resolution (e.g., 22 μm in the *xy* plane), and exceptional adaptability. Both conductive and nonconductive materials, can be 3D printed. Recently, stereolithography (SLA) 3D printing technique have been explored to fabricate pH solid MN arrays (Figure 5c).^33^ Continuous pH monitoring in plants was achieved by integrating polyaniline (PANI) sensing films specifically engineered for hydrogen ion detection. The MN arrays were utilized to observe plant diurnal rhythms over a duration of approximately 4 days and to assess pH variations induced by drought stress. As previously noted, the repeatability and conductivity of PANI sensors may decline with time, mostly due to changes in the polymer composition as well as structural/morphological transformations.^21^ Interestingly, the implementation of ionophore-based polymer membranes can markedly decrease the response time of pH MN sensors to roughly 3 s,^92^ in contrast to the 45–60 s reported for PANI pH electrodes.^21,33^

## Hollow Microneedles

Another type that can be used as sensors is the hollow MNs, designed with a hole at the tip and hence, a hollow capacity that can be empty for fluid extraction or be filled with the sensing element(s). For plants, this type of MNs have been fabricated by using 3D printing.^29^ Although, as far as we know, there are not any hollow MN reported for ions monitoring until the time of this writing, the experience already gained with their employment in sap extraction and analysis of other analytes can be interesting to be adapted for ions. In the first case, an absorbent paper is attached to a hollow MN patch, and the collected sap is then analyzed with a commercial screen-printed electrode (e.g., to detect hydrogen peroxide). Although this design provides convenient on-site measurements, it is limited to single-use analysis.^29^

Alternatively, a sensing element consisting of a modified wire can be embedded within a 3D-printed hollow MN.^30^ In this configuration, the hollow MN primarily serve as a casing that protects the electrodes from damage during penetration into the tough tissues of plants. This strategy enables continuous and reliable plant monitoring. Moreover, the sensor electrodes can be independently manufactured, calibrated, and stored off-site. When required for field applications, they can be conveniently assembled and deployed. Overall, hollow MNs not only exhibit exceptional sensing capabilities but also hold significant potential for the targeted delivery of combined therapeutics. A noteworthy study highlights the possibility of precisely administering agrochemicals within the vascular systems of plants, enabling localized treatment of specific tissues to combat pathogens.^93^ Moreover, plant growth regulators, such as gibberellic acid, can be accurately applied using MNs.^94^ Importantly, this dual functionality (sensing + therapeutics delivery) paves the way for the development of closed-loop systems for nutrient management.^95^ In such systems, sensors can detect ion deficiencies in plants and trigger the release of essential nutrients, both functions through MN devices.

## Microelectrodes

A planar microelectrode may also be used for monitoring the internal physiology of plants, despite not being explicitly categorized as a MN nor conforming to the traditional three-dimensional MN configuration. Researchers shaped the tip of a polyimide (PI) electrode into a triangular form with an optimized thickness (Figure 6).^31^ This design provides sufficient mechanical strength, enabling it to efficiently penetrate the epidermis layer and embed within the stem. In this scheme, the sensing element is firmly attached to the substrate. The implanted part of the microelectrode was 3 mm wide and 3 mm long, and an incision (2 mm) must be made on the tomato stem. Since plants do not exhibit sensitive pain responses comparable to humans, it seemed acceptable at the current stage of research if the procedure does not inflict significant, irreversible harm on the plant.

**Figure 6:**
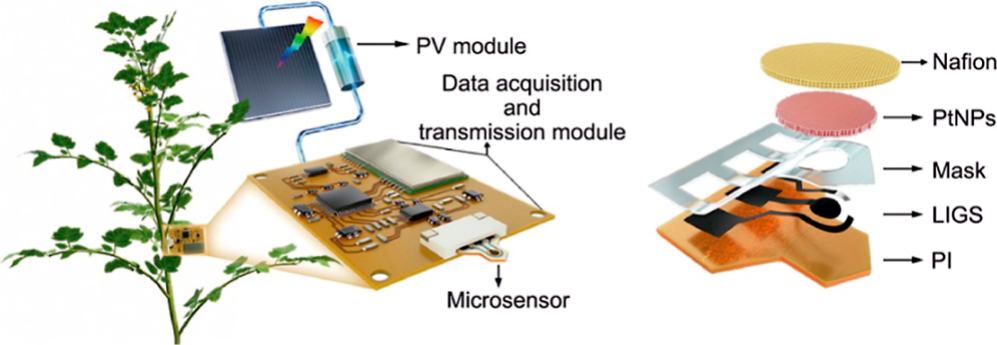
Microelectrodes for in vivo plant monitoring.^31^ Reproduced from ref 31. Copyright 2024 Elsevier.

## Analytical Potential of Potentiometry as Redout in Microneedle ION Sensors

Through the integration of electrochemical techniques like potentiometry, MN sensors can provide real-time ion monitoring in plants. Interestingly, the selection of the sampling rate in the electronic component allows the intervals for data acquisition providing measurements with millisecond-level precision. Moreover, the miniature design of MN ion sensors ensures high spatial resolution, making it possible for precise detection of ions in specific, localized regions within plants. Compared to many existing detection methods, which are often prohibitively expensive, ion sensing on MN devices offer a cost-effective and scalable alternative, ensuring affordability for farmers and feasibility for large-scale agricultural deployment. Their simplicity further enhances their utility, eliminating the need for complex sample preparation and providing an intuitive “insert-and-measure” approach.

Potentiometric sensors support multimodal detection, that is, allowing simultaneous measurement of multiple ion concentrations.^92^ This capability is particularly valuable in applications such as disease prediction in plants, where comprehensive ion profiling significantly improves the accuracy of disease risk assessments. Typically, the cause of a specific disease in a particular plant species can be attributed to various ion imbalances. By transcending the constraints of conventional techniques, which are generally limited to single-ion detection, MN sensors offer a revolutionary approach for enhancing smart agriculture.

Considering parameters such as selectivity, sensitivity, equipment complexity, and others, potentiometric sensing emerges as the most effective approach for ion detection. This technique involves measuring the open circuit potential (OCP), also referred to as the electromotive force (EMF), between a reference electrode (RE) and a working electrode (WE). The measurement provides valuable insights into the ionic activity of a specific analyte, which is governed by the Nernst equation.^96^ It is a low-energy method particularly suitable for portable and field-deployable systems.^96–98^ With a low limit of detection (LOD) and a broad linear range of response (LRR), potentiometric sensing enables accurate quantification of ion concentrations across multiple orders of magnitude. Furthermore, it is exceptionally well-suited for real-time and continuous monitoring applications (e.g., healthcare and environmental monitoring).^99,100^

Ion-selective electrodes (ISEs) are indispensable platforms in potentiometric sensing. Currently available ISEs are capable of measuring ion concentrations within a logarithmic activity range of 10^–6^–10° (i.e., from 1 μM to 1M). This range is sufficient to cover the major ion levels found in plants under both healthy and pathological conditions. These devices rely on ion-selective membranes (ISMs) that exhibit specific responsiveness to target ions. Typically, the ISM is composed of ion-exchangers and ionophores, with this latter playing a critical role in the selective binding and transport of target ions across the membrane.

In potentiometric solid-state sensors, the format to be used in MNs, an ion-to-electron transduction layer is needed between the ISM and the electrode surface is needed. This layer serves as an essential interface that converts ionic signals into measurable electronic outputs. Commonly utilized materials include conductive polymers, such as polypyrrole and polyaniline, as well as carbon-based nanomaterials like carbon nanotubes and graphene. Conductive polymers are particularly advantageous due to their high ion permeability and biocompatibility, facilitating efficient ion transport and stable signal transduction. Conversely, carbon-based nanomaterials are valued for their large surface area and superior conductivity, which contribute to enhanced sensitivity and signal stability.^99,100^

## Current Challenges and Prospects When Translating Potentiometry into the Microneedle Format

### Figures of Merit

In potentiometric measurements, what is measured is ion activity, which must be converted to ion concentration. The commonly used Debye–Hückel approximation requires knowledge of the sample ionic strength; however, accurately determining the ionic strength of plant sap poses significant challenges. Ionic strengths exhibit considerable variation across different plant species and within the same species at various developmental stages. To address this variability, one potential approach is to consult existing literature to estimate ion concentrations specific to plant species, and growth stages in question. In any case, this method is susceptible to errors due to the unpredictability of the growth environment. Alternatively, an estimation can be achieved by preanalyzing sap samples collected from targeted plants, allowing for the direct measurement of ionic strength. In another direction, but only demonstrated at a very fundamental level, Gao et al. introduced the concept of utilizing a self-referencing Ag/AgI pulstrode as a reference element for the direct measurement of monovalent anionic species without ionic strength influence.^100,101^ While this approach presents a promising solution, further investigation is necessary to advance this concept or to explore alternative strategies for practical implementation. On the other hand, the pulstrode procedure will limit the data acquisition frequency.

The sensitivity and selectivity of ISEs are crucial parameters for the precise determination of specific ions in complex sample matrices, such as sap. In principle, the response slopes for ions with varying valence states are determined by the Nernst equation. At a temperature of 25 °C, the theoretical slope is approximately 59.2 mV/decade for monovalent ions (e.g., Na^+^, K^+^, Cl^–^), 29.6 mV/decade for divalent ions (e.g., Ca^2+^, Mg^2+^), and 19.7 mV/decade for trivalent ions (e.g., Al^3+^, Fe^3+^). A decrease in these slope values results in a reduced sensitivity, thereby increasing the probability of significant errors. Notably, to palliate this effect, the improvement of the detection accuracy by increasing the Nernst slope has been proposed. Nonetheless, this method requires the separation of the WE and RE into distinct solution environments.^96^ This prerequisite poses considerable challenges for the engineering of MN sensors, which are designed to enable continuous monitoring within a unified sample environment. Concerning selectivity, any ionophore intrinsically has a certain degree of selectivity for interfering ions. For instance, in the analysis of ion distributions within plants, Na^+^ ions are typically found at much lower concentrations than K^+^ ions. Targeting Na^+^, the ionophore will exhibit a certain level of binding affinity also for K^+^ and hence, elevated concentrations of K^+^ may interfere with Na^+^ measurements, thus compromising the overall accuracy. An appropriate selectivity study and response algorithms and protocols accounting for possible interferences may be necessary in certain cases. Fortunately, these have been already developed for other applications and could be easily translated to the MN configuration.^102^

Another important aspect is the inherent conditioning and calibration procedures of the MNs prior use. Indeed, the right calibration protocol is yet a controversial subject for MNs measurements.^88^ In analogy to animal models, some approaches include sensor calibration both before and after in-planta measurement. This dual calibration not only enables more accurate analyte quantification but also serves to evaluate the impact of tissue penetration on sensor performance, correct potential signal drift, and assess phenomena such as biofouling and changes in the sensor–tissue interface over time (e.g., wound healing). Typically, these calibrations are performed in buffered solutions and assuming negligible matrix effect. Despite a better choice would be using an artificial version of the corresponding biological fluid, there is a key limitation for in-planta measurements related to the variation in sap composition among different plant compartments (i.e., xylem and phloem) and species. Consequently, not only is the selection of an appropriate calibration matrix challenging in this context, but also the design of a universal protocol for in-planta measurements. Thus, if establishing a reliable calibration-free approach is already one of the major challenges in the wearable field, it becomes even more demanding in the context of in-planta wearables.

### From the Lab to Field

Deploying potentiometric MN sensors in natural environments poses certain challenges connected to temperature fluctuations, humidity, and physical disturbances. Temperature changes can impact sensor precision, as the Nernst equation predicts a 0.1 mV shift in EMF for every 1 °C. Thus, the MN may necessitate robust temperature compensation, especially under extreme conditions. In addition, biofouling, which occurs when microorganisms, or other biological materials accumulate on the sensor surface, can significantly degrade sensor performance over time. To mitigate this, protective coatings and surface treatments that prevent biofilm formation may be needed for long-term measurements. On the other hand, environmental stressors such as UV radiation, wind, and dust can degrade sensor performance, while rainfall and humidity may compromise ISMs and electronics. Protective coatings and sealed designs are essential to ensure durability and reliability in field measurements. Additionally, the sensors must withstand mechanical stresses from plant growth and motion without frequent recalibration. Overall, to transition MN ion sensors from the lab to field, innovations in material science and sensor design are critical to maintaining accuracy and functionality in dynamic outdoor conditions. Also, advances in the design of electronics to be coupled with the sensors are necessary for recording and transmitting the chemical information.

Another important aspect is the biocompatibility of the MN sensing platform. In contrast to biomedical devices, in-planta sensor investigations have prioritized the assessment of the analytical performances over the potential phytotoxic effects. In fact, phytotoxicity tests have often been limited to wound healing and vast physiological changes, while issues such as cellular toxicity, tissue damage, and long-term stress responses remain underexplored.

## Other Crucial Aspects

### Plant Terminology

Ambiguity and inconsistency in plant science terminology often involve significant challenges in the interpretation of findings and integration of knowledge across studies. Terms such as “sap,” “apoplast,” “epidermis,” and “cuticle” are often variably defined, yet commonly used within the same context, making it difficult to compare and synthesize research outcomes. For example, the term “sap” is widely used to describe any plant fluid, although its composition and function significantly differ between xylem sap and phloem sap.^103^ Similarly, “apoplast” is sometimes narrowly defined as nonliving spaces, whereas in other instances, it is extended to include specific cellular interfaces.^104^ At present, these terms are frequently used interchangeably in the literature about plant sensors. Moreover, “epidermis” and “cuticle” are occasionally treated as synonymous, despite being distinct layers of plant tissue with unique and specific functions.^105,106^ These inconsistencies highlight the urgent need for clear and standardized definitions to reduce misinterpretation and enhance the coherence of research in plant science.

### Implications in Agriculture and Plant Health

Intelligent agricultural practices have been demonstrated to improve productivity, preserve resources, and reduce environmental consequences, yielding beneficial outcomes for the economy, ecology, and workforce. Nonetheless, quantifying their precise influence is difficult, while the technological and innovation needs are clear. Climate change has diminished worldwide agricultural productivity by 21% since the 1960s.^107^ Substantial losses, totaling $27 billion in U.S. crops from 1991 to 2017, can be attributed to climate change.^108^ Droughts in Europe result in annual losses of €9 billion, with forecasts suggesting a potential yield reduction of up to 50% in southern Europe by 2050.^109^ The Mediterranean region may experience a loss in agricultural output exceeding 10%, highlighting the necessity for adaptive methods.^109^ Moreover, a recent FAO analysis disclosed that $10 trillion in concealed environmental, social, and health expenses are attributable to the existing food and agricultural systems.^110^

In such a context, wearable plant sensors, recognized as a leading emerging technology by the World Economic Forum, are anticipated to improve plant health and productivity.^111^ Truly, the correlation between ion concentrations and the health, quality, and resilience of plants is essential for contemporary agriculture. The concentrations of ions, especially Ca^2+^ and K^+^, are crucial factors influencing the freshness, maturity, and decay resistance of plants and fruits. Identifying fluctuations in these ion concentrations facilitates accurate assessment of fruit deterioration phases and quality evaluation. This information can improve postharvest management and storage methods to preserve crop quality.^111^

Monitoring the flow of ions in plant stems, particularly near the roots and leaves, offers a comprehensive insight into nutrient absorption and physiological health. Root-based ion flows, involving ions like K^+^, Ca^2+^, and NO_3_^–^, reflect nutrient absorption efficiency and soil health.^112^ Stable ion transport patterns are indicative of optimal root function and adequate nutrient availability, while disruptions can signify soil deficiencies, water stress, or root damage.^113^ Effectively, an early detection can guide prompt soil management and irrigation strategies to avert crop losses. Then, in leaves, ion flows (especially of K^+^) directly influence stomatal behavior, gas exchange, and photosynthetic activity. High, consistent ion flow supports vigorous photosynthesis and active growth, whereas irregular patterns may indicate environmental stress, such as drought, salinity, or nutrient imbalance.^114^ Detecting these variations allows for early mitigation strategies, such as adjusting irrigation, applying fertilizers, or managing salinity, to protect crop yields.

Stressors such as drought, salinity, and pathogen infections can induce substantial alterations in ion transport, as plants initiate defensive strategies, including stomatal closure and localized ion accumulation. Monitoring these changes offers an immediate insight into the plant’s stress response prior to the manifestation of apparent symptoms. This prediction ability enables farmers and researchers to undertake remedial measures, thereby diminishing crop susceptibility to environmental challenges.^115^ The dynamics of Na^+^ are particularly crucial in relation to salt stress, an increasing issue for agriculture in saline soils. Assessing Na^+^ absorption and translocation from roots to stems and leaves under simulated saline stress conditions elucidates initial reactions to elevated salinity. This technology provides exceptional insights into Na^+^ accumulation and transport channels, permitting the proactive identification and control of salt stress prior to significant damage. These developments represent considerable potential for enhancing crop resilience in adverse settings.

### From the Lab to Crops: Analyzing the Device Costs beyond the Sensing Part

The global market size of smart agriculture is expected to grow significantly between 2025 and 2030, reaching USD 43.37 billion by 2030.^116^ Despite the potential and progress of MNs sensors for plants, significant advances are still required to move up the technology readiness level for its commercialization. To achieve a final product, the developed sensor should couple with an electronic module that records and transmits the signals. Most of the studies focus on the technical performance and potential applications of the MNs sensors overlooking this aspect, which significantly impacts the technical and economic feasibility as well as their practical adoption in real-world scenarios.

Many parts of the plants or even the plant itself (when it is small) are fragile and can be damage due to the weight of the sensors and the device.^20^ Thus, with the intention of maintaining plant integrity, extreme miniaturization of the entire sensor, including the electronics, is required. Indeed, reducing size compromises not only the cost, due to a more complex design, but also the features of the final device such as autonomous operation or data acquisition time. To put it in perspective, the cost for a PCB can increase from less than $20 (reported as low-cost miniaturized potentiometric design) to $500 in the most advanced miniaturized multilayers PCB.^117^ While it should be considered that low-volume or prototype runs are generally more expensive per unit compared to mass production, alternative actions can be adopted. For example, the sensors can act as an independent element decoupled from the rest of the measurement unit which can be placed in alternative locations, such as the roots, allowing bigger electronics and wire connections that simplify the system. Consequently, the cost can be minimized, and the implementation less technologically complicated by separating the sensor from the electronics, with the sensor being the only disposable component.

However, advances in nanotechnology and microfabrication techniques make us optimiztic about the possibility of a miniaturized final device for plant applications. Among other factors, power integration and data transmission are the most critical aspects in achieving a miniaturized final device. Power constraints arise from the need for small batteries that must efficiently support sensors, microcontrollers, and wireless modules. These devices require careful optimization to balance power consumption and longevity, as frequent recharging is a limiting factor for implementation. Emerging energy harvesting technologies, such as thermoelectric generators, biofuel cells, or photovoltaic cells, for self-powered sensors are transforming the wearable device landscape by enabling sustainable and autonomous operation without traditional batteries. The implementation of wireless charging systems can be another alternative.^118,119^ Data transmission, on the other hand, faces challenges related to bandwidth, reliability, security, and energy consumption. Wireless protocols like bluetooth low energy (BLE) and near-field communication (NFC) are designed for low power consumption but can struggle with transmitting large amounts of real-time data, especially in environments with high interference. As an intermediate alternative to fully decoupling the sensor and the electronics, these devices can also be integrated with other sensors or energy sources, such as soil sensors. This approach allows for a more flexible system design, where the sensor module could remain compact while being supported by additional components to enhance functionality.

### Commercial Readiness of MN Sensors: Key Barriers

Based on the aspects discussed in this perspective, this section presents an overview of the critical barriers that still stand in the way of bringing MN sensors to market. Moving from proof-of-concept devices to commercial systems requires not only addressing engineering challenges but also overcoming key chemical and sensor-related constraints. Briefly, key challenges include sustained performance in biological environments, resistance to biofouling, and the development of calibration-free strategies that maintain accuracy without the need for frequent recalibration. From an engineering perspective, one persistent issue is the high cost of compact, integrated electronics. A potential strategy to mitigate this involves decoupling the sensing interface from the processing unit, which may reduce per-device costs and facilitate scalable deployment.

Beyond technical considerations, the lack of dedicated regulatory frameworks for in planta devices further complicates commercialization. Ongoing concerns regarding safety, environmental impact, biodegradability, and disposal continue to generate uncertainty for manufacturers and investors. Finally, demonstrating a clear return on investment remains an open challenge, not due to a lack of potential, but because comprehensive, large-scale validation in agricultural environments is still limited. Establishing quantitative evidence that links MN sensor deployment to agronomic or economic benefits will be essential for their widespread adoption, particularly in resource-constrained agricultural settings.

## Conclusions

Microneedle sensors represent a transformative advancement for in-planta ion monitoring, offering a minimally invasive, real-time method for detecting critical ionic changes within plant tissues. This innovative technology surpasses traditional techniques by enabling direct access to internal plant compartments with minimal tissue disruption and heightened spatiotemporal resolution. The capability to continuously monitor ions within living plants provides unprecedented insights into plant physiology, stress responses, and nutrient uptake. Despite existing challenges, such as improving sensor durability, ensuring reliable performance in field conditions, and addressing biocompatibility issues, ongoing progress in microneedle design and potentiometric sensing are prone to propel this field forward. With further improvement, microneedle ion sensors hold the potential to become indispensable tools in plant phenotyping and agricultural innovation, ultimately contributing to sustainable crop management and resilience in the face of environmental change.
